# Perspectives of work readiness among Australian health students trained during the COVID-19 pandemic

**DOI:** 10.1186/s12909-024-06044-3

**Published:** 2024-09-27

**Authors:** Tegan Podubinski, Belinda Jessup, Melissa Kirschbaum, Jodie Bailie, Susan Heaney, Lyndal Sheepway, Lisa Bourke

**Affiliations:** 1 Department of Rural Health, The University of Melbourne, 38 Green Street, Wangaratta, VIC 3676 Australia; 2 Centre for Rural Health, University of Tasmania, E Block, Newnham Campus, Launceston, TAS 7250 Australia; 3 University Centre for Rural Health, The University of Sydney, 61 Uralba Street, Lismore, NSW 2480 Australia; 4 Department of Rural Health, The University of Newcastle, 20 Highfields Circuit, Port Macquarie, NSW 2444 Australia; 5 La Trobe Rural Health School, La Trobe University, Edwards Road, Flora Hill, VIC 3550 Australia; 6 Department of Rural Health, The University of Melbourne, 49 Graham St, Shepparton, VIC 3630 Australia

**Keywords:** Allied health, Graduate preparedness, Work readiness, Medicine, Nursing, Placements, Rural health workforce, Transition to practice

## Abstract

**Background:**

To explore perspectives of work readiness, including readiness to work rurally, among health students trained in Australia during the COVID-19 pandemic.

**Methods:**

Participants were allied health, medicine, and nursing students in the later years of their degree (third, fourth or final year of an undergraduate entry to practice degree, or second year of postgraduate entry to practice degree), where training is clinically immersive. These students had completed a University Department of Rural Health facilitated rural and remote placement between January 2021 and October 2022. They participated in a cross-sectional online survey (*n* = 426), comprising Likert-scale questions. Interested survey respondents participated in a semi-structured interview (*n* = 34). Multiple logistic regression was conducted to examine the predictors of work readiness within the survey, and interview data was analysed via reflexive thematic analysis.

**Results:**

Among survey respondents, 69.7% felt they would be ready to be a health practitioner when the time came to graduate and 71.8% felt clinically prepared to work in a rural location. Concerns about having developed enough clinical skills on placements to competently practice on graduation and being able to continue studying their course during the pandemic were both predictive of work readiness and feeling clinically prepared to work rurally. Four themes reflecting factors impacting work readiness were developed from interview data: (1) *‘I’d estimate probably a 20–30% reduction in face-to-face handling practice over the course of all of my placements’* encompassed student concerns regarding the collective impact of cancelled placements and lower patient attendance at healthcare facilities on clinical skill development; (2) *‘Two and a half years of sitting behind a computer’* related to student experiences of superficial learning and cohort disconnectedness due to online course delivery and loss of on campus simulations; (3) ‘*I’ll still need like a lot of support in my grad year’* related to students recognising the need for support and supervision post-qualifying to bridge the gaps in their learning; and (4) ‘*We are the COVID nurses’* encompassed student recognition of skills gained including communication skills, competence with technology and telehealth, knowledge of infection control, and work readiness skills (e.g. adaptability and resilience) as a result of training during the global pandemic.

**Conclusions:**

Universities can support work readiness during pandemic circumstances by fostering clinical skills development through continuation of quality placement experiences and face-to-face curriculum delivery. Although health graduates trained during the pandemic are likely to have a range of additional work ready skills, health services will need to proactively support their transition into the workforce in the coming years.

**Supplementary Information:**

The online version contains supplementary material available at 10.1186/s12909-024-06044-3.

## Introduction

The education and training of health professionals in Australia is highly regulated to ensure a quality health workforce. University training programs must meet national accreditation standards encompassing comprehensive curriculum and clinical placement requirements, which aim to produce competent, high-quality, work-ready graduates [1]. The Australian Government also encourages access to quality rural and remote training experiences via its network of University Departments of Rural Health (UDRH) and Rural Clinical Schools (RCS) to ensure continued growth of the health workforce outside of metropolitan cities [2]. UDRHs are academic centres based in rural and remote Australia focused on health and education training and research. They receive funding to provide training for a particular number of placement weeks (ranging from approximately 3000 to 6000 placement weeks) to nursing, allied health, and medical students from any university in Australia. RCSs are focused on providing high-quality, long-term (usually one year or more), rural and remote health training opportunities for approximately 25% of Commonwealth supported medical students, from the same university. Through these programs, students are exposed to a variety of practice settings, which may help to prepare them for rural and remote practice by building relevant skills and networks [3]. For the purposes of this paper, the term rural and remote encompasses all areas outside Australia’s major cities [4], and the focus is on placements facilitated by UDRHs.

The concept of readiness to practice in the healthcare professions, that is, having the skills and attributes required to enter the health workforce, has not been universally described [5, 6]. Numerous terms have been used synonymously, including readiness, preparedness, fitness or suitability for practice or work, and clinical competence [5]. Attributes or characteristics used to describe work readiness vary and include not only academic knowledge and clinical competence, but also organisational acumen and personal characteristics such as resilience, flexibility and stress management, social intelligence or critical skills like teamwork, effective communication, independence, conflict management, personal insight and self-awareness, and professionalism [7]. Furthermore, a range of methods have been used to assess health graduates’ practice preparedness, graduate self-perceptions [8, 9], the perceptions of supervisors [10, 11] and measurement via instruments [7, 12]. This makes comparisons between studies difficult.

The transition from university to clinical practice can be an overwhelming and stressful time for newly graduated health professionals [9, 13], particularly for graduates commencing work in rural and remote areas, who face the additional challenges of geographical isolation and resource shortages [14]. Furthermore, rural and remote clinicians can be expected to manage highly complex and diverse caseloads as sole practitioners [15]. Factors that have been shown to facilitate work readiness include knowledge and clinical skills, quality teaching, clinical placements and support from supervisors, family and peers [9]. In addition, preparedness for rural and remote practice is known to be enhanced when students have undertaken rural and remote placements during their training [8, 16]. Participation in rural and remote placement programs offers students valuable opportunities to acquire a broad spectrum of skills, including problem-solving, adaptability, self-confidence, autonomy, and interprofessional practice. These skills can enable students to become ‘work-ready’ rural and remote clinicians [17, 18]. Conversely, barriers to health workforce readiness include heavy academic workloads, limited knowledge of clinical governance and health systems [9], , work stressors, working long hours [10], insufficient practical experience, and lack of skills in conflict, time and stress management [19].

The widespread disruption to the delivery of health education and routine healthcare services during the COVID-19 pandemic, affecting both metropolitan and rural and remote settings, has been extensively described, with existing research providing insight into the impact of the pandemic on student education and experiences, the transition to online learning, changes to teaching methods and placements [20, 21], missed opportunities to practice clinical skills [22] and negative effects on mental health and wellbeing [23, 24]. These impacts to education and training have the potential to alter the enablers of work readiness [9]. However, to date, there has been limited research regarding the impact of the pandemic on health graduates’ readiness for practice, and particularly on readiness for rural and remote practice. Initial studies suggest a reduced level of readiness; however further research has been recommended [25–28]. To address this identified gap in the literature, this study explores the perspectives of health students in the clinically immersive final years of their study, as related to work readiness, and the factors that predict their perceived readiness to practice, both as a health practitioner and as a health practitioner in a rural and remote location. It contributes to a larger study conducted amidst the ongoing COVID-19 pandemic, which examined impacts to placements, clinical training, and graduate preparedness from the perspectives of health students who had a UDRH-facilitated rural and remote placement during 2021 and 2022 [29]. UDRH-facilitated placements are seen to be a crucial component in addressing the health workforce maldistribution; they are designed to ensure that graduates are well-prepared for rural and remote practice, increasing the likelihood that they will choose to work in rural and remote areas post-graduation [2]. By focusing specifically on these students, this study aims to identify insights into students’ views on work readiness and the factors that shaped their preparedness during the COVID-19 pandemic.

## Method

### Design

This study uses both quantitative data from the online survey and qualitative data from the semi-structured interviews. The larger study utilised a parallel convergent mixed methods design to concurrently collect quantitative and qualitative data using a cross-sectional online survey and semi-structured interviews [29].

### Ethics approval

This research received ethical approval from the University of Tasmania Human Research Ethics Committee (Project ID: 27664). Other participating universities (The University of Melbourne, The University of Newcastle, La Trobe University, The University of Queensland, The University of Western Australia, Flinders University and James Cook University) obtained ethical approval or registration from the own institutions, as appropriate.

### Recruitment

Given the aims of the broader body of work, all UDRHs across Australia were contacted to purposefully recruit health students who had completed a rural and remote (Monash Modified Model 2–7 [30]) placement between 1 January 2021 and 31 October 2022. This time frame was chosen as it was after the initial phase of the pandemic in 2020 which saw many rural and remote placements cancelled or changed to virtual experiences [20]. In 2021-22, many student placements continued, although activities, learning outcomes and student experiences were sometimes altered by the pandemic [29]. For this study, students were invited to participate via an email invitation that included a link to the online survey, distributed by the 16 UDRHs. To maximise the response rate, two follow-up emails were sent, one a week after the initial email and another two weeks later. Participation in the survey was voluntary and completion implied consent. Survey respondents who were in the later years of their degree (third, fourth or final year of an undergraduate entry to practice degree, or second year of postgraduate entry to practice degree) were invited to leave their contact details at the end of the survey if they were interested in participating in an interview to discuss their experiences in more detail. A total of 115 provided their contact information at the end of the survey. Of these, 60 were randomly selected to be contacted to be interviewed; 20 did not respond, five declined to be interviewed and one failed to attend their scheduled interview time and could not be contacted again to reschedule. Random selection enabled all students to have the opportunity to be chosen, preventing specific regions, disciplines, from influencing the selection process. The remaining 34 students were interviewed individually via Zoom. Interviews ranged from 21 to 79 min in length (average 45 min).

### Data collection

Survey data were collected using LimeSurvey hosted by the University of Tasmania. The full survey comprised a total of 34 questions, collecting demographic information and asking students about the ongoing impact of the COVID-19 pandemic on their clinical training and planned rural and remote placement(s) using a mix of forced choice, Likert-scale and free text questions. A subset of these questions were included for analysis in this study, relating specifically to course progression, placement opportunities, the development of clinical skills on placement, and graduate preparedness (see Appendix A).

Interviews were conducted individually via Zoom, by one of four trained members of the project team (BJ, JB, LS, SH). Interviews were organised so that the interviewer was not affiliated with the UDRH who facilitated the student’s placement. Students were asked about their scheduled rural and remote placement, changes or impacts to placements because of the ongoing pandemic, and other questions regarding perspectives of clinical training and work readiness (Appendix B). All interviews were audio-recorded and transcribed verbatim. Transcripts were returned to interviewees to allow for any amendments to be made before being deidentified by allocating a numeric code to denote participant number and removing the names of university and placement locations.

### Data analysis

Survey and then interview data were analysed separately by the first and second authors (TP, BJ). Survey data were analysed using IBM SPSS Statistics for Windows, version 27 (IBM Corp) using a combination of descriptive and inferential statistics. For descriptive analyses, categorical variables are presented as frequency (*n*) and percent (%). Multiple logistic regression was conducted to examine the predictors of readiness to be a health practitioner upon graduation and clinical preparedness for rural and remote practice. Variables rated on a five-point Likert scale (see Appendix A) were recoded into a dichotomous variable to allow for the regression; strongly disagree, disagree and neither agree or disagree were recoded into 0 = did not agree, while agree and strongly agree were recoded into 1 = agree.

Interview data were subjected to an iterative process of reflexive thematic analysis, as described by Braun & Clarke [31]. Familiarity with transcripts was achieved by multiple readings by members of the research team (TP, BJ, MK, JB, LS, SH). Interview data related specifically to (rural and remote) work readiness was then inductively coded using NVivo (version 12) independently by two members of the research team (BJ, MK), with relevant initial codes collapsed into preliminary subthemes and then themes as coding progressed. Subthemes and themes were modified and then agreed upon through regular consensus discussions with the broader research team to ensure they accurately reflected student perspectives. Verbatim quotations were used to exemplify the themes identified. Results from the quantitative and qualitive analysis were integrated in the discussion Sect. [32].

## Results

### Survey results

A total of 857 students responded to the online survey, resulting in an overall response rate of 12.1%. However, 322 responses did not provide usable data for analysis (304 non-responses and 18 responses were incomplete). Of the 535 complete responses received, seven were excluded due to the respondent providing data concerning a placement that occurred in 2020, and eight were excluded as the respondent listed their placement location as a metropolitan capital city, leaving 520 useable responses. Of these, 426 were identified as students in the later years of their degree and were therefore the focus of analysis in this study.

Most survey respondents were female (*n* = 344, 80.8%) and studying undergraduate degrees (*n* = 330, 77.5%). Around half (*n* = 221, 51.9%) were under the age of 25 years (Table 1). Only nine (2.1%) respondents identified as Aboriginal and/or Torres Strait Islander, while exactly half (*n* = 213, 50.0%) indicated they had a rural background. Over half (*n* = 252, 59.2%) were completing their studies through blended methods (e.g. both face to face and online components). Responses were received from students studying in all states and territories of Australia; however, most respondents were from Victoria (*n* = 142, 33.3%). Half of students (*n* = 215, 50.5%) began studying their course prior to the pandemic.

**Table 1:** Demographic characteristics of survey respondents (*n* = 426)

Characteristics	Allied Health *n* = 207 *n* (%)	Nursing *n* = 160 *n* (%)	Medicine *n* = 59 *n* (%)	Total *n* = 426 *n* (%)
**Gender**				
*Male*	31 (15.0)	25 (15.6)	23 (39.0)	79 (18.5)
*Female*	173 (83.6)	135 (84.4)	36 (61.0)	344 (80.8)
*Non-binary*	3 (1.4)	0 (0.0)	0 (0.0)	3 (0.7)
**Age**				
*Under 25 years*	125 (60.4)	62 (38.8)	34 (57.6)	221 (51.9)
*25 to 34 years*	65 (31.4)	41 (25.6)	22 (37.3)	128 (30.0)
*Over 35 years*	17 (8.2)	57 (35.6)	3 (5.1)	77 (18.1)
**Aboriginal and/or Torres Strait Islander**				
*Yes*	6 (2.9)	1 (0.6)	2 (3.4)	9 (2.1)
*No*	200 (96.6)	156 (97.5)	57 (96.6)	413 (96.9)
*Undisclosed*	1 (0.5)	3 (1.9)	0 (0.0)	4 (0.9)
**Rural Background**				
*Yes*	89 (43.0)	93 (58.1)	31 (52.5)	213 (50.0)
*No*	118 (57.0)	67 (41.9)	28 (47.5)	213 (50.0)
**Course Level**				
*Undergraduate*	144 (69.6)	151 (94.4)	35 (59.3)	330 (77.5)
*Postgraduate*	63 (30.4)	91 (5.6)	24 (40.7)	96 (22.5)
**Course Commencement**				
*≤* *2018*	52 (25.1)	13 (8.1)	21 (35.6)	86 (20.2)
*2019*	73 (35.3)	36 (22.5)	21 (35.6)	129 (30.3)
*2020*	56 (27.1)	83 (51.9)	14 (23.7)	153 (35.9)
*2021*	24 (11.6)	25 (15.6)	3 (5.1)	52 (12.2)
*Not reported*	2 (1.0)	3 (1.9)	0 (0.0)	5 (1.2)
**Mode of Study**				
*Blended*	122 (58.9)	109 (68.1)	21 (35.6)	252 (59.2)
*Face-to-Face*	65 (31.4)	21 (13.1)	38 (64.4)	124 (29.1)
*Online*	20 (9.7)	30 (18.8)	0 (0.0)	50 (11.7)
**University Location**				
*Australian Capital Territory*	2 (1.0)	1 (0.6)	0.0 (0.0)	3 (0.7)
*New South Wales*	45 (21.7)	21 (13.1)	14 (23.7)	80 (18.8)
*Northern Territory*	0.0 (0.0)	5 (3.1)	1 (1.7)	6 (1.4)
*Queensland*	42 (20.3)	16 (10.0)	16 (27.1)	74 (17.4)
*South Australia*	16 (7.7)	9 (5.6)	3 (5.1)	28 (6.6)
*Tasmania*	4 (1.9)	30 (18.8)	12 (20.3)	46 (10.8)
*Victoria*	65 (31.4)	64 (40.0)	13 (22.0)	142 (33.3)
*Western Australia*	33 (15.9)	14 (8.8)	0.0 (0.0)	47 (11.0)

#### Course progression and graduation, clinical learning opportunities, and work readiness

While most students agreed that they had been able to continue studying their health course during the pandemic (*n* = 394, 92.5%), just over a quarter had concerns about graduating on time due to the pandemic (*n* = 123, 28.9%) (Table 2). A quarter of students felt that the pandemic had lessened their opportunities for rural placements (*n* = 105, 24.6%), with a similar proportion indicating they had not had enough placement experience during their course due to the pandemic (*n* = 120, 28.2%). Just over half would have liked more placement time during their course (*n* = 236, 55.4%), and almost half would have liked more rural placements during their course (*n* = 189, 44.4%). One in five students did not feel that they had developed enough clinical skills on placements to competently practice after graduation (*n* = 92, 21.6%). Slightly over two-thirds of students felt that they would be ready to be a health practitioner when the time came to graduate (*n* = 297, 69.7%), and a similar proportion felt clinically prepared to work in a rural location after they graduated (*n* = 306, 71.8%). Therefore, there were concerns about exposure to clinical practice and clinical readiness to graduate.

**Table 2:** Frequency (%) of students in agreement survey statements by total sample and discipline (*n* = 426)

	Allied Health *n* = 207 *n* (%)	Nursing *n* = 160 *n* (%)	Medicine *n* = 59 *n* (%)	Total *n* = 426 *n* (%)
**Course progression and graduation**				
*I have been able to continue studying my health course during the pandemic*	195 (94.2)	142 (88.8)	57 (96.6)	394 (92.5)
*I have concerns about graduating on time due to the pandemic*	43 (20.8)	78 (48.8)	2 (3.4)	123 (28.9)
**Clinical learning opportunities**				
*The pandemic has lessened my opportunities for rural placements*	51 (24.6)	41 (25.6)	13 (22.0)	105 (24.6)
*I do not feel that I have had enough placement experience during my course due to the pandemic*	52 (25.1)	59 (36.9)	9 (15.3)	120 (28.2)
*I would have liked more placement time during my course*	132 (63.8)	80 (50.0)	24 (40.7)	236 (55.4)
*I would have liked more rural placements during my course*	108 (52.2)	60 (37.5)	21 (35.6)	189 (44.4)
**Work readiness**				
*I do not feel that I have developed enough clinical skills on placements to competently practise when I graduate*	26 (12.6)	58 (36.3)	8 (13.6)	92 (21.6)
*I feel that I will be ready to be a health practitioner when the time comes to graduate*	156 (75.4)	91 (56.9)	50 (84.7)	297 (69.7)
*I feel clinically prepared to work in a rural location after I graduate*	157 (75.8)	98 (61.3)	51 (86.4)	306 (71.8)

#### Predictors of work readiness

The multiple logistic regression analysis found that for each outcome, the full model containing all predictors was statistically significant (Table 3). Concerns about having developed enough clinical skills on placements to competently practice on graduation (OR = 0.18, CI = 0.10–0.33, *p* = < 0.001; OR = 0.29, CI = 0.17–0.48, *p* = < 0.001) and being able to continue studying their course during the pandemic (OR = 4.58, CI = 2.00-10.49, *p* = < 0.001; OR = 3.37, CI = 1.54–7.41, *p* = < 0.002) significantly predicted whether a student agreed that they felt ready to be a health practitioner when the time came to graduate and whether a student agreed that they felt clinically prepared to work in a rural location after they graduated (respectively). Each model correctly classified 77.2% and 73.7% of cases respectively.

**Table 3:** Multiple logistic regression analysis predicting work readiness (general and rural)

	I feel that I will be ready to be a health practitioner when the time comes to graduate^^^		I feel clinically prepared to work in a rural location after I graduate^+^	
	Odds ratio (95% confidence interval)	*P*	Odds ratio (95% confidence interval)	*P*
Origin			1.38 (0.87–2.20)	0.17
Discipline *Allied Health (0)*		0.07		0.04*
*Nursing (1)*	0.68 (0.41–1.14)	0.15	0.68 (0.41–1.12)	0.13
*Medicine (2)*	1.84 (0.77–4.39)	0.17	2.02 (0.85–4.78)	0.11
Mode of study *Blended (0)*		0.62		0.87
*Face-to-Face (1)*	1.17 (0.65–2.11)	0.59	0.98 (0.55–1.72)	0.93
*Online (2)*	1.41 (0.67–2.99)	0.37	0.83 (0.41–1.66)	0.60
I do not feel I have had enough placement experience during my course due to the pandemic *Agree (1)*	0.64 (0.37–1.12)	0.12		
The pandemic has lessened my opportunities for rural placements *Agree (1)*			0.77 (0.46–1.29)	0.32
I do not feel that I have developed enough clinical skills on placements to competently practice when I graduate *Agree (1)*	0.18 (0.10–0.33)	< 0.001*	0.29 (0.17–0.48)	< 0.001*
I have been able to continue studying my course during the pandemic *Agree (1)*	4.58 (2.00–10.49)	< 0.001*	3.37 (1.54–7.41)	0.002*
Constant	1.04	0.93	1.19	0.70

* Statistically significant *p* < 0.05

^^^ R^2^ = 19.8% (Cox & Snell), 28.1% (Nagelkerke). Model X^2^ (7, *N* = 426) = 94.18, *p = <* 0.001

^+^ R^2^ = 11.5% (Cox & Snell), 16.6% (Nagelkerke). Model X^2^ (8, *N* = 426) = 52.19, *p* = < 0.001

### Interview results

Of the 34 students who were interviewed, 11 (32.4%) were studying nursing, six (17.6%) medicine, and the remaining 17 (50.0%) various allied health disciplines including occupational therapy, speech pathology, physiotherapy, pharmacy, psychology, paramedicine, social work, dietetics, exercise physiology, public health, and health information management. Interviewees were predominantly female (*n* = 21, 61.8%) and over 25 years of age (*n* = 25, 73.5%).

#### Overall perceptions of work readiness

There were mixed responses by interviewees regarding overall work readiness. Some students described feeling confident and ready to graduate as a health professional. These students felt that they had learned as much as they could as a student and were ready to enter the workforce to continue their learning journey.

‘I am rip-roaring to go. I have been ready for the last six months.’ (Interviewee #4, medicine).

However, others described feeling less skilled than they ought to when considering they were graduating soon.

‘I do feel a bit more undercooked than I otherwise might.’ (Interviewee #13, psychology).

Some students made the point that it was impossible to know how much more prepared they would have felt if they had not trained during a global pandemic.

‘I do feel mostly prepared. I mean it’s hard to know how prepared I would have been if COVID hadn’t have happened.’ (Interviewee #20, medicine).

Students also shared mixed feelings regarding readiness for rural practice. Some students felt confident that they could meet the demands of graduate employment in a rural setting.

‘I know that I have the experience now to be able to do it… I feel like by seeking the rural placements that I’ve had, I’m feeling pretty comfortable with the situation that I’ll be in next year in [rural area].’ (Interviewee #4, medicine).

However, others were doubting that their skills and abilities were suitable for initial employment in a rural location.

‘I would love to stay out rural and remote, but just given this year and how some things have been, and with limited support, I don’t feel as confident in myself to be out here.’ (Interviewee #9, midwifery).

#### Factors impacting work readiness

Following thematic analysis, four key themes and eleven subthemes were identified relating to student perspectives of impacts to work readiness (Table 4).

**Table 4:** Key themes and subthemes relating to factors impacting work readiness

Key Theme	Subthemes	Meaning
‘Reduction *in face-to-face handling practice over the course of all of my placements’*	1) Opportunities for clinical skill development on placements 2) Proactively bridging the gaps	Encompassed student experiences of work readiness resulting from opportunities for hands on clinical learning on placements. Loss of opportunities resulted from cancelled placements and the collective reduction of patient presentations across placements.
*‘Two and a half years of sitting behind a computer’*	1) Online learning 2) Loss of on-campus simulation 3) Social disconnection	Encompassed student experiences of cancelled face-to-face lectures and practical sessions, the rapid transition to online course delivery and the condensing of course content into shorter periods of time. Collectively, this contributed to students feeling a superficial level of theoretical and clinical knowledge, and loss of social connectedness with their student cohort.
*‘I’ll still need like a lot of support in my grad year…’*	1) Support during transition to practice 2) Support in metropolitan versus rural locations	Encompassed student beliefs of the need for additional support and supervision during transition to practice, to bridge the gaps in theoretical knowledge and clinical skills. Students perceived that supportive roles were mostly available in metropolitan settings given the workforce shortages and solo positions often available in rural locations.
*‘We are the COVID nurses…*’	1) Verbal communication 2) Technology 3) Infection Control 4) Work readiness skills	Related to students recognising that training during the unique pandemic context had strengthened some graduate preparedness skills including verbal communication, technology and telehealth, knowledge of infection control procedures, problem solving, and adaptability and resilience.

##### ‘I’d estimate probably a 20–30% reduction in face-to-face handling practice over the course of all of my placements’

###### **Opportunities for clinical skill development on placements**

Students recognised that work readiness was interlinked with opportunities for clinical skill development on placements. For some students, they reflected that they felt ready to graduate following placements that had allowed them to develop autonomy in delivering healthcare. These students suggested they were acting independently and functioning essentially as a new graduate on placements which gave them an indication that they were adequately skilled and ready for practice.

‘I do feel ready because when I was on my last, probably my last few placements, so my rural one as well, by the last few weeks I was really running things as my own and having like support, but kind of taking my own clients and everything so I feel ready in that aspect.’ (Interviewee #21, occupational therapy).

Conversely, other students described feelings of unpreparedness, associated with lost and impacted opportunities to develop clinical skills on placements. Some students described losing entire placements at the height of the pandemic in 2020 which they felt left them behind clinically for the remainder of their course.

‘Definitely a couple of my earlier placements were quite impacted and one quite significantly, and as a result I felt like I started this final placement with less skill than I otherwise might and less competence than I otherwise might. … I think I could have benefitted more from those earlier placements if it weren’t for COVID because I think they were better placements. And then that meant when I arrived at this last placement, that frankly wasn’t a very good placement, I arrived feeling a bit behind and I don’t feel like it contributed as much, I don’t feel like I’ve made that up at that placement. I don’t feel like I’ve bridged that gap.’ (Interviewee #13, psychology).

Students also reflected on the collective reduction in patient presentations to healthcare facilities across placement experiences throughout the pandemic that they believed further left their clinical skills underdeveloped.

‘My first placements were sort of January to May 2021, and we were actually in and out of lockdown. I’d be on placement and then the next day we’re back in lockdown and everything’s been moved to telehealth. We were fortunate in that we were still seeing inpatients in hospital, but I’d estimate probably a 20–30% reduction in face-to-face handling practice over the course of all of my placements compared to what would have been in 2019.’ (Interviewee #6, physiotherapy).

###### **Proactively bridging the gaps**

Some students were fortunate to have had supervisors and other health professionals who were cognisant of the impacts to student learning throughout the pandemic and who were motivated to help students make up for lost opportunities in their clinical learning. These supervisors and health professionals spent additional time generating simulated activities to support learning on placements and fostered opportunities to build clinical skills.

‘Some facilitators were incredible and actually cared about what type of skills you’d have when you entered nursing and they would joke and say, ‘I don’t just want you to be a Zoom nurse because the patient’s going to be a real patient, not a Zoom patient, and you need to get used to the needles and how to do a bandage’. They would go through simulations and the skills that I gained, wound care, cannulation, I’ve nailed all those skills because of the placement.’ (Interviewee #11, nursing).

Other students reflected that they had taken it upon themselves to build opportunities to bridge gaps in their clinical learning by gaining employment in the healthcare sector, volunteering to gain extra clinical experience, and seeking extra opportunities and specific patients or settings whilst on placement to help address missing clinical skills.

‘At the very end of the placement, I asked my supervisor if I could do another assessment and report just to consolidate everything, and she was like, ‘okay, if it’s just you, you can go.’ So we did an evening visit just quickly in and out. We made sure to go for a resident that wasn’t particularly vulnerable and that helped consolidate my skills.’ (Interviewee #1, speech pathology).

##### ‘Two and a half years of sitting behind a computer’

###### **Online learning**

Some students reflected that work readiness had been impacted by changes in the way course content and learning experiences were delivered in response to the pandemic. First, the rapid transition from face-to-face lectures to online delivery left some students feeling that their theoretical knowledge was superficial. Students recognised that much of the richness in their learning of course content prior to the pandemic occurred because of in-person group discussion which was lost in the virtual delivery of lectures and tutorials.

‘When COVID wasn’t actually even known at all, we had a lot more face-to-face and you learnt more by being in the group and bouncing ideas off each other, not so constricted by like a Zoom group kind of discussion where people feel as if they can’t really, may be reluctant to ask, but they do feel kind of removed… And you learn, once again, it’s having that kind of brainstorming and being able to see things practically and to actually share information, to share content… I feel as if with the whole COVID pandemic, that’s what really has been impacted and it impacts your confidence.’ (Interviewee #34, nursing).

Although other students described the return of blended models of learning, comprising some face-to-face teaching supported by online learning, students described the inherent challenge of absorbing large amounts of course content in such a short space of time.

‘Restrictions and that have dropped now but the university is still kind of acting as if COVID’s still around and it hasn’t gone back to the face-to-face like they did before COVID. They’re still working on us doing all our content within three weeks on campus and then the rest is just all online at home. It’s really hard to learn all that stuff in three weeks.’ (Interviewee #30, nursing).

###### **Loss of on-campus simulation**

Students also described changes to the delivery of on campus simulation activities. This included a range of tutorials and practical sessions which would have seen students gain real life experience of how to undertake clinical tasks, such as injections, wound care, splinting, drug preparation and taping. Students described that due to pandemic restrictions, classes were cancelled or converted to online sessions resulting in lost opportunities to engage with people and healthcare equipment in real life.

‘It’s great to have all this knowledge on how the body works and how diseases work but if you can’t apply it, what’s the point? And two and a half years of sitting behind a computer … I can talk about a broken arm and a broken leg [but] when was the last time I put a leg splint on? … When I actually do it, it’d be for the first time, that’s the scary concept, so I’m going to look like a goose for a good few months.’ (Interviewee #7, paramedicine).

When restrictions permitted, practical sessions were rescheduled; however, students described that these sessions were condensed over shorter time periods and with fewer people due to social distancing restrictions which meant less time and opportunity to practice the range of skills. Students reflected that the amount of time devoted to simulations was inadequate to allow for deep learning and confidence with practical skill development.

‘When we did do stuff in class at uni, we were shown once if we were lucky how to do something and that’s where a whole lot of us before we went to placement were like, ‘I feel like I am not safe.’ … I certainly voiced that when I went to one of my placements at the [regional hospital] last year, that I felt really nervous about giving my first intramuscular injection. We’d practiced all that on bits of rubber and now I was about to do it on a living human. I’m fine now, but that was quite frightening that we only had one go in the lab and that was it. One go.’ (Interviewee #10, nursing).

###### **Social disconnection**

Students also described that due to the transition of course learning to predominantly online delivery, that they missed opportunities to meet other students and establish friendships and networks within their student cohort. Consequently, students described feeling socially isolated, with limited professional relationships on which to call on to help support the transition to practice.

‘I’m going to graduate in two weeks, and I don’t know half of my cohort because we just haven’t spent any proper time together.’ (Interviewee #26, exercise physiology).

##### ‘I’ll still need like a lot of support in my grad year…’

###### **Support during transition to practice**

Given the various impacts to both theoretical and clinical learning, many students were feeling vulnerable about commencing work as a health professional, as described by a nursing student: ‘I think everyone’s got impostor syndrome’ *(*Interviewee #34).

Some students reflected that while they felt they could safely practice, they needed good support structures to help bridge the gaps in their learning and confidence before working autonomously. Students therefore spoke about the importance of seeking supported graduate positions that would allow them to continue their learning journey and develop confidence under supervision.

‘I’ll still need like a lot of support in my grad year … I guess it’s just a matter of if I don’t know something, asking and making sure that I’m not going to do it wrong.’ (Interviewee #30, nursing).

Others spoke about the importance of receiving clinical supervision and guidance as a normal part of ongoing learning experiences.

‘I think there are areas, and this is always going to be the case, I think there are areas where I’d still require more supervision. So, for example, eating disorders is an area that’s quite highly specialized that a lot of people don’t feel confident in upon graduating. And so there would be, there’ll be certain areas that were I pursuing those areas, I would seek additional training or additional supervision.’ (Interviewee #15, Psychology).

##### Support in metropolitan versus rural locations

With all students having completed a rural placement, many reflected on their experiences that rural employment often involved working in isolation, with limited supervision and in the context of workforce shortages. Consequently, although many students indicated that they eventually wanted to work rurally after graduating, they felt that it was unsuitable for them to undertake rural employment immediately post-qualifying. Many students described wanting to spend a period of time in a busier, metropolitan setting to ensure adequate support and gain experience, skills and confidence to then be able to move to a rural setting in the future. However, some students shared that they were willing to consider rural employment on the proviso that adequate supervision and support structures were available.

‘Some people say you learn so much more in a rural area, where some people say you learn more in [metropolitan city]. And I think, for me, the support was a big one, and I was witness to the lack of support that was available in rural areas for the clinicians and so I’m in [metropolitan city] for a bit to get that support and extra education, before I head back to a country place.’ (Interviewee #21, occupational therapy).

#### ‘We are the COVID nurses…’

##### Verbal communication

A nurse, Interviewee #33, aptly stated that ‘*I think we are the COVID nurses’*. This statement captured a sentiment that while training during a global pandemic had resulted in many challenges, it had also fostered unique strengths that were characteristic of this cohort of students. Students recognised that their communication skills were stronger than anticipated due to engaging with people via technology, having to increase verbal instruction due to social distancing and having to develop strategies to communicate while wearing a mask.

‘I know my verbal instruction has improved out of sight. I’m a lot better now at, I guess, getting people to do things for themselves and explaining the why behind them.’ (Interviewee #26, exercise physiology).

##### Technology

Students also described that they inherently became proficient in using technology and telecommunications both to facilitate their learning and to provide clinical care on placements throughout the pandemic. Students reported that videoconferencing platforms such as Zoom became utilised to support their learning and as such, had extensive practice in presenting and engaging with others on this platform. Using telehealth to provide clinical care was identified as a specific bonus of training during the pandemic by some. Students felt that without the pandemic, this would not have been a learnt skill.

‘I’d never actually had a telehealth appointment or performed telehealth prior to university and obviously the pandemic, and from what I gather it wasn’t going to be a very big part of what our course would have been. We probably wouldn’t have hardly touched it. So I’ve gone from maybe a couple of hours over three years to now having done 50–60 hours worth of telehealth on placements. I mean I’ve done entire weeks where all five days, all six appointments, were all telehealth.’ (Interviewee #6, physiotherapy).

##### Infection control

Students described that the pandemic had also strengthened their knowledge of infection control procedures and the use of personal protective equipment. Students were also acutely aware of the COVID-19 virus itself and pandemic management strategies because of assignments and placements that were embedded in the context of COVID-19.

‘You’re probably a lot more conscious of personal protective equipment like gowning and gloving. Obviously before COVID, you may never have done that, or like done it once in a blue moon, whilst now it’s like second nature.’ (Interviewee #5, medicine).

##### Work readiness skills

Finally, students described that a benefit of training during the pandemic was learning to adjust to a rapidly changing environment. Students described that they experienced the rapid pivot to online learning and changes to placement experiences which helped them learn to be adaptable, resilient in the face of challenges, and proactive in solving problems.

‘I guess having to navigate things on your own a bit more, obviously being online was challenging, well it was for me anyway, still trying to cover things where you would normally just ask in class for some help right then and there, you kind of just, you either don’t get a response or have to wait like for days or something for an answer. Like, you just kind of got to learn a bit more how to solve things on your own, which I think is good. It’s a good thing.’ (Interviewee #14, pharmacy).

## Discussion

The present study aimed to investigate perspectives of work readiness among health students who trained in Australia during the COVID-19 pandemic and completed rural and remote placements as part of their education. Around 70% of students agreed that they felt ready to be a health practitioner when the time came to graduate, and that they felt clinically prepared to work in a rural location after they graduated. These are encouraging findings and suggest that despite the limitations on training and education due to the pandemic, students are largely emerging work ready [33]. However, almost one third of students expressed concern that they did not feel ready to be a health professional, while a similar proportion did not feel prepared to work in a rural setting specifically. The results of this study suggest that at least some of this is due to loss of clinical knowledge, skills and networks, all of which have been identified as important enablers in work readiness [9]. Regardless of perceptions of readiness to work, student feedback has provided key insights into a range of strategies that can be adopted by universities and health services to support work readiness among medical, nursing, and allied health graduates, especially those trained during pandemic circumstances.

### Lesson one: ensuring opportunities for clinical skills development

Our study found that student experiences during clinical placements played a significant role in shaping their perceived work readiness; a finding consistent with the literature [9]. This finding was anticipated given clinical placements are a cornerstone of healthcare education, offering students invaluable opportunities to bridge the gap between theory and practice, develop clinical skills, and gain confidence in their abilities [9]. Understandably, some students drew confidence and readiness from placements that fostered autonomy and independence, giving them a tangible indication that they possessed the skills and competence required for graduate practice [9]. These results reinforce the positive contribution of some placements undertaken during the pandemic period toward fostering work readiness amongst health students [33].

However, other students in this study faced significant challenges due to disruption to their clinical experiences caused by the pandemic. Students described that the loss of clinical placements, reduced patient encounters, and limited hands-on learning opportunities collectively impacted their perceptions of readiness as they prepared to enter the healthcare workforce. This underscores the pivotal role of hands-on clinical experiences in shaping health students’ self-assessment of their preparedness for practice post-qualifying [33–35]. The fact that having enough placement opportunities (including rurally) was not predictive of students’ perceived readiness to practice, however, indicates that it is not so much the number of placement opportunities that impact readiness to practice, but the extent to which these placements develop hands-on skills. This is further backed by the lesser odds of readiness to practice when students felt they had not developed enough clinical skills on placement to competently practice upon graduation. This supports the findings of Smith and colleagues [34] who reported that the most common response from graduate nurses as to how to better prepare for graduate practice was more practice in patients’ overall hands-on management. As such, educational institutions, healthcare facilities and supervisors should continue to prioritise and optimise clinical placements to ensure that students acquire the foundational skills required for their future practice [34].

Our study revealed a group of students who, despite their work readiness, highlighted the significant effort they had invested in reaching this point. These students demonstrated a proactive approach to addressing gaps in their knowledge and clinical experience and actively seeking opportunities to enhance their readiness for the healthcare workforce. Their accounts shed light on the dedication and resilience required to navigate the challenges posed by the COVID-19 pandemic and draw attention to positive strategies to facilitate work readiness, including volunteering to gain extra clinical experience, securing paid employment in the healthcare sector, and actively seeking additional clinical exposure or specific patient encounters during their placements. Furthermore, students acknowledged the support and understanding of supervisors and other healthcare professionals in facilitating their readiness, with some supervisors going to great lengths to provide additional guidance, opportunities, and mentorship to assist health students to acquire the necessary clinical competencies whilst on placement. Recognition of skill deficits and a commitment to addressing these among supervisors may therefore ensure continuation of work ready graduates during pandemic circumstances [36, 37].

### Lesson two: preserving education delivery

The quantitative results of this study also demonstrated that students who were able to continue studying their course during the pandemic had greater odds of perceiving themselves as being ready to practice as healthcare professionals, and in a rural location. This highlights the importance of uninterrupted education, even in challenging circumstances like a global pandemic, to promote work readiness [35]. Although universities and educators in Australia and worldwide have explored innovative approaches and adapted curriculum delivery accordingly to accommodate pandemic disruptions [38], student satisfaction with these adaptations has varied [35, 39–41]. In the qualitative results, students in this study described that online course delivery approaches impacted their engagement with course materials and their cohort, something that was valued from face-to-face delivery methods [35, 40]. Students also reflected on the loss of on campus clinical simulations which both prepared them for placements, and ultimately, for employment [35, 40, 42]. Shorey and colleagues [39] recommended that hybrid models of curriculum delivery, including online content supported by face-to-face components, as the way forward for health education; a model supported by this study if work readiness is to be promoted [43].

Interestingly, our qualitative results also observed that in addition to the impact on theoretical learning, students emphasised the impact of adapted curriculum delivery on social aspects of their education. Due to the transition to online course delivery, students described disrupted opportunities to meet and establish friendships and networks with their peers. As a result, they were concerned they had limited professional relationships on which to rely for guidance and support during the transition into the healthcare workforce. This is consistent with the findings of Malau-Aduli et al. [9] who found that social supports from peers, friends, family and university staff is a key enabler of work readiness. Although social challenges have been reported in the literature in response to online learning [39, 40], universities may have underestimated the impact of cohort disconnectedness for students navigating the stressful period of applying for jobs and transitioning into the workforce for the first time.

### Lesson three: providing support for workforce transition

Our findings revealed a common theme among students: the perceived desire for additional support and supervision within the workplace as they transition into their first professional role post-qualifying. These findings do not appear to be unique to the pandemic context, with literature identifying that graduates trained prior to the pandemic also expressed hesitancy about work readiness and confidence to undertake initial employment after graduation [34, 44]. As some students reflected in this study, it is impossible to ascertain how much more prepared they would have felt if they hadn’t trained throughout the pandemic.

This study observed that the desire for supported roles led to a preference for metropolitan rather than rural employment, a finding mirrored elsewhere [45]. While students harboured the aspiration to serve as healthcare professionals in rural areas, many believed they lacked the clinical skills and confidence necessary to immediately undertake rural employment upon qualification. These students described a strategic approach to their career trajectories, articulating a preference for starting their careers in busier metropolitan settings to gain essential experience, skills, and the confidence required for healthcare practice before considering rural practice. Their rationale behind this choice was grounded in a realistic assessment of the demands and expectations of rural employment gained during their rural placement experiences. Indeed, research confirms that these pre-emptive concerns are valid, with early career health professionals who transition to rural settings often facing inadequate support, fuelled by a lack of staff and resource shortages [15, 45–47].

However, a proportion of students in this study did feel well-prepared for rural practice as new graduates. These students attributed their readiness to the positive rural placement experiences, which provided exposure to the nature of rural employment and instilled a sense of competence and confidence in their ability to fulfill the roles expected of them in rural settings. Positive rural placement experiences are therefore a critical mediating factor in building rural workforce through encouraging early career healthcare professionals who may embed themselves in rural communities longer term [48]. UDRHs, who are responsible for facilitating healthcare placements in rural settings, need to ensure that students are allocated to healthcare settings which provide quality support and supervision and develop graduate level competencies and independence [48]. These demonstrative placement experiences may help challenge student beliefs that only metropolitan settings can offer the level of support they are seeking after qualifying.

### Lesson four: harnessing pandemic related skill strengths

While students were reflective of potential skill deficits, they also were quick to acknowledge that training during the pandemic had facilitated additional unforeseen competencies that supported work readiness. Consistent with the literature, students described having enriched communication skills [33], technological proficiency [22, 33, 49], knowledge and practice in infection control protocols [33, 35], and a range of work readiness skills including adaptability, resilience, and problem solving [33, 35]. These additional skills and attributes represent valuable assets that will not only enhance their readiness for professional practice [33], but also instil the capacity to thrive in dynamic and unpredictable healthcare environments and provide the skills and mindset needed to navigate the complex realities of healthcare delivery in the post-pandemic world. Ironically, these skills would serve students well for transition to practice specifically in rural settings which often require graduates to exhibit greater autonomy and resourcefulness in response to resource constraints [15, 46, 47]. Universities and placement sites need to continue to provide opportunities to develop these skills moving forward.

### Limitations and future research

It is important to acknowledge that individuals involved in this study would have encountered varying pandemic-related impacts, depending on where they were located, when they started their degree, and when and where they undertook their placement/s. Thus, it is difficult to determine the level to which such contextual factors have shaped participants’ work readiness. Additionally, students may have had a range of simulated learning experiences which were not captured in this study which may have contributed to perceptions of work readiness. Furthermore, the cross-sectional nature of the research design limits our ability to draw causal conclusions. Longitudinal studies that extend on the questions in this study could provide more robust evidence for the factors impacting student’s preparedness to practice. Additionally, the study relied on self-reported data, which may introduce bias. It should also be noted that although a response rate has been calculated, this might be an overestimation as some UDRHs may not have advised how many students were emailed. Further, the response rate was low, suggesting that these results may not be generalisable to all students who completed a rural and remote placement across Australia during the study period. It is also worth noting that the qualitative analysis is based on less than 10% of the students invited for survey, again potentially impacting generalisability. Furthermore, perceptions of graduate preparedness were explored among students in the later years of their degree, including those who may not have been in their final year. It may be that the perceptions of students where graduation was imminent differed to those who still had some time remaining in their degree. Finally, there was no comparison with students in non-pandemic years; it may be that that students in their final years are often unsure about readiness to practice due to levels of confidence rather than skill level or impact of the pandemic.

Given the above, future research could explore the transition of health students who completed training during the pandemic, especially into rural and remote settings. Understanding their transition experiences, levels of support, job satisfaction, and the quality of care they provide will help understand how to improve pandemic health training. It would also be beneficial to examine how health professional training programs can better prepare students for entering rural and remote practice immediately upon completing their studies, and how rural and remote organisations can provide stronger support to new graduates. Furthermore, investigating the specific components of clinical skills development that contribute to perceived readiness to practice, including in rural and remote areas, along with the role of placements in cultivating these skills, could help inform the design of effective educational interventions.

### Conclusion

This study highlights the nuanced landscape of work readiness among Australian health students trained during the COIVD-19 pandemic. While the majority expressed readiness for professional practice, a subset were concerned about their transition to professional practice, especially in rural and remote settings, emphasising the need for targeted supports. Universities and healthcare services can address these concerns by prioritising clinical skills development and supervision, maintaining face-to-face curriculum delivery, and fostering social connectedness among student cohorts. Further, it is important to continue this learning during pandemics to bring on the next cohort of health professionals and ensure work readiness. Looking forward, although health graduates in the immediate post-pandemic years are likely to have a range of additional work ready skills, the healthcare workforce must anticipate the need for enhanced support, mentorship, and guidance for early career professionals, particularly in rural and remote settings, to ensure their successful transition into the post-pandemic healthcare landscape. Overall, new health graduates are seeking to join supportive workplaces, and health organisations who want to recruit new graduates will need to ensure the resources are there to actively nurture and develop them. By proactively addressing these challenges, stakeholders can better equip the next generation of healthcare professionals to navigate the evolving demands of healthcare delivery in the wake of the pandemic.

## Electronic supplementary material

Below is the link to the electronic supplementary material.

Supplementary Material 1

## Data Availability

The datasets analysed during the currently study are not publicly available to protect study participant privacy, as per ethical approvals. Requests for the dataset can be made to the corresponding author, noting the need for appropriate ethical approvals.
