# Supplementary material for: Perspectives of work readiness among Australian health students trained during the COVID-19 pandemic
Source: BMC Med Educ. 2024 Sep 27;24:1053. doi: 10.1186/s12909-024-06044-3 (PMC11428983; doi:10.1186/s12909-024-06044-3)
Supplement: Supplementary file 1 — Supplementary Material 1 [file 12909_2024_6044_MOESM1_ESM.docx]

# Appendix A: Survey Questions

1. **What gender do you identify with?**
   - Female
   - Maile
   - Non-gender binary
2. **How old are you?**
   - <25 years
   - 25-34 years
   - 35< years
3. **Do you identify as Aboriginal and/or Torres Strait Islander?**
   - Yes
   - No
   - Do not want to say
4. **Do you identify as having a rural background? (Rural background is defined as having lived whilst growing up for five years continuously or ten years in total in a location outside of a capital city, Geelong, Wollongong, or Newcastle)**
   - Yes, I do identify as having a rural background
   - No, I don’t identify as having a rural background
5. **Is your health course an undergraduate or postgraduate degree?**
   - Undergraduate
   - Postgraduate
6. **In what year did you commence your health course?**
7. **How are you currently completing your health course?**
   - Face to face
   - Online
   - Blended (e.g. both face to face and online components)
8. **What state or territory is the University you are enrolled in?**
   - Queensland
   - Victoria
   - New South Wales
   - South Australia
   - Northern Territory
   - Western Australia
   - Tasmania
   - Australian Capital Territory
9. **What health discipline/s are you currently studying? (Please tick all that apply)**
   - Medicine
   - Nursing
   - Midwifery
   - Aboriginal/Torres Strait Islander Health
   - Audiology
   - Chiropractic
   - Dental Hygiene/Therapy
   - Dentistry
   - Oral Health
   - Dental Prosthesis
   - Diagnostic Radiography
   - Dietetics
   - Exercise Physiology
   - Medical Laboratory Science
   - Medical Radiation Science
   - Nutrition & Dietetics
   - Occupational Therapy
   - Optometry
   - Orthotics and Prosthetics
   - Osteopathy
   - Paramedicine
   - Pharmacy
   - Physiotherapy
   - Podiatry
   - Psychology
   - Public Health or Health Promotion
   - Social Work
   - Speech Pathology
   - Other (please specify)
10. For each of the following statements, please identify if you strongly disagree, disagree, neutral, agree or strongly agree (Third, fourth, final year students only):
    - I have been able to continue studying my course during the pandemic
    - I have concerns about graduating on time due to the pandemic
    - I do not feel I have had enough placement experience during my course due to the pandemic
    - The pandemic has lessened my opportunities for rural/remote placements
    - I do not feel that I have developed enough clinical skills on placements to competently practise when I graduate
    - I feel that I will be ready to be a health practitioner when the time comes to graduate
    - I would have liked more placement time during my course
    - I would have liked more rural/remote placements during my course
    - I feel clinically prepared to work in a rural/remote location after I graduate
    - I would like to work in a rural/remote location after I graduate

# Appendix B: Interview Questions

1. **Perhaps we can start with a little bit of information about you:**
   - What is your discipline of study?
   - What year of study are you in?
   - What university do you study at?
   - Do you normally live in a rural or remote location while studying?
   - Gender?
   - Under or over 25 years of age?
   - Do you identify as having a rural background?
2. **I understand that you were scheduled to undertake a rural or remote placement sometime since January last year. Can you tell me about that placement and how it came about?**
   - How far away was the placement from where you live while you study?
   - What sort of placement was it? (i.e. hospital based, service learning, area of study, etc.)
   - Did you choose the placement?
   - Did you choose to go to a rural or remote location?
   - Did you receive any financial support to undertake placement?
   - Did you have to be vaccinated to undertake placement? How did you feel about that?
3. **Did you go on the rural/remote placement as planned?**
   - Was it your choice to (not) go on placement? Why/why not?
   - Were you happy to (not) go on placement during COVID-19? Why/why not?
   - Can you describe your experience of the decision to (not) go on this placement?
   - What are the implications for you resulting from (not) going on this placement?

**If the student did not do any placement, skip to Q.6**

1. **Can you tell me about your experience of undertaking your rural or remote placement during the pandemic?**
   - What was the placement like?
   - Was the placement what you expected?
   - Did you get out of the placement what you wanted?
   - What do you feel that you missed out on, on placement?
   - Did you learn new skills that you didn’t expect?
   - Can you describe the accommodation you stayed in?
   - Can you tell me about the supervision your received?
   - Did you feel supported during placement?
   - Did you feel that you had the opportunity to immerse yourself in the rural/remote community where your placement was based?
2. **Did COVID-19 change your placement in any way? If so, in what ways?**
   - Where placement was located?
   - Tasks undertaken?
   - Supervision?
   - Contact with patients/clients?
   - Contact with students?
   - Use of technology?
   - Accommodation?
   - Support provided?
   - What did you think of these changes?
3. **Did you have any concerns about your health and safety on placement? Can you tell me about these concerns?**
   - Did you feel at risk at any time?
   - Did you feel that your safety was well considered?
   - Did you feel that you would have liked to have taken more risks clinically?

**(Questions 1-5 can be repeated if student has done more than one rural or remote placement in**

**the last two years)**

1. **Do you have any concerns about graduating soon?**
   - Do you feel ready to graduate?
   - Do you feel that you have developed enough clinical skills to safely practise as a health professional?
   - Are there clinical skills that you feel you are lacking?
   - Do you feel that you have had enough clinical training during your course?
   - Would you feel skilled enough to work in a rural or remote location?
   - Do you feel there are skills you haven’t been able to develop as a result of training during the pandemic?
   - Are there skills that you are graduating with that you didn’t expect as a result of training during the pandemic?
2. **Do you have any interest in working in a rural or remote location after you graduate?**
   - Have you always wanted to (or not wanted to) work in a rural or remote area? Why/why not?
   - Have your recent or other rural training experiences (or lack of) influenced your choice?
   - Has the pandemic influenced where (location wise) you see yourself working after you graduate?
